# Language comprehension warps the mirror neuron system

**DOI:** 10.3389/fnhum.2013.00870

**Published:** 2013-12-17

**Authors:** Noah Zarr, Ryan Ferguson, Arthur M. Glenberg

**Affiliations:** ^1^Department of Psychology, Arizona State UniversityTempe, AZ, USA; ^2^Department of Psychology, University of Wisconsin-MadisonMadison, WI, USA

**Keywords:** language comprehension, mirror neurons, neural adaptation, motor system, embodied cognition

## Abstract

Is the mirror neuron system (MNS) used in language understanding? According to embodied accounts of language comprehension, understanding sentences describing actions makes use of neural mechanisms of action control, including the MNS. Consequently, repeatedly comprehending sentences describing similar actions should induce adaptation of the MNS thereby warping its use in other cognitive processes such as action recognition and prediction. To test this prediction, participants read blocks of multiple sentences where each sentence in the block described transfer of objects in a direction away or toward the reader. Following each block, adaptation was measured by having participants predict the end-point of videotaped actions. The adapting sentences disrupted prediction of actions in the same direction, but (a) only for videos of biological motion, and (b) only when the effector implied by the language (e.g., the hand) matched the videos. These findings are signatures of the MNS.

## Introduction

Language comprehension is a simulation process: A sentence is understood by using linguistic symbols to drive neural systems of action (Rizzolatti and Arbib, 1998; Fischer and Zwaan, 2008), perception (Meteyard et al., 2008), and emotion (Havas et al., 2010) into states homologous to those created by actual experience in the described situation. For example, to understand a sentence such as “You give the pencil to Henry,” a listener uses her motor system to simulate the action of giving (e.g., moving the arm away from the body while the hand is performing a precision grip), and uses her visual system to simulate the visual characteristics of a pencil.

Simulation accounts (e.g., Glenberg and Gallese, 2012) suggest that the motor system plays a constitutive role in meaning. That is, activity within the motor system is, itself, part of the meaning of the sentence. If correct, then there should be a bi-directional causal relation between motor activity and language comprehension: Changing the motor system should causally affect language comprehension, and changing language comprehension should causally affect the motor system. In both cases it is because language comprehension and motor activity are one and the same thing. Several experiments (discussed later) have demonstrated such bi-directional links using EEG. Here we focus on whether the mirror neuron system (MNS) may be a playing a role in these links.^1^

Previous work (Glenberg et al., 2008) demonstrated half of the bi-directional link, namely that adapting the motor system through repeated literal action affects language comprehension. In those experiments, participants moved beans from one container to another for about 15 min. For half of the participants, the direction of movement was from a location close to the participant to one farther away, and for the other participants the direction of movement was from a far container to a near container that is, toward the body. This repeated action adapts the motor system (Classen et al., 1998). But does repeated action affect language comprehension? The data suggest an affirmative answer: After repeated action in the Away direction, participants were slower to comprehend sentences describing action Away (e.g., “You give Alice the pizza”), and after repeated action Toward, participants were slower to comprehend sentences describing action Toward (e.g., “Alice gives you the pizza”). Why is there a slowing? One possibility is that the relevant action control system is fatigued. A second possibility is that the action control becomes specialized for the repeated movement (e.g., moving a bean using a power grip). Then, when the action control system is called upon to simulate a different movement (e.g., an open-handed movement used to pass a pizza), fewer neural resources are available.

In the work reported here, we demonstrate the other half of the bi-directional link. Participants read a block of sentences all of which described action of a particular sort, for example, transfer away using the hand. On the assumption that language comprehension of action sentences requires a simulation using the motor system, then repeatedly comprehending sentences of the same sort should adapt the motor system much as does repeatedly moving beans.

But, how are we to demonstrate that the motor system has been adapted by the language task? We took advantage of the putative fact that the MNS (Rizzolatti and Craighero, 2004), a component of the motor system, plays a role in both language and action perception. The MNS is active both when an animal engages in action and when the animal perceives a conspecific take similar action (Rizzolatti and Craighero, 2004). MNS activity has been linked to language on theoretical grounds (Rizzolatti and Arbib, 1998), using imaging techniques (Aziz-Zadeh et al., 2006), and using behavioral techniques (Glenberg et al., 2008).

When the MNS is engaged, it facilitates prediction of biological motion. For example, an observer's eyes anticipate the location of an actor's hand when the actor is stacking blocks. But when the actor's hand is invisible, so that the blocks appeared to move on their own (i.e., non-biological motion), the eyes lag the blocks; that is, prediction is impaired (Flanagan and Johansson, 2003).

Because the MNS is multi-modal, adapting it through repeated action (Classen et al., 1998) should affect both action perception (Cattaneo et al., 2011) and language comprehension (Glenberg et al., 2008). Here we document the role of the MNS in language comprehension by using the complementary procedure. Namely, if comprehension is a simulation process that uses the MNS, then repeated comprehension of sentences should adapt the MNS. We measure the effects of adaptation using a visual prediction task.

Much like Flanagan and Johansson (2003) our experiment used a manipulation of biological and non-biological motion. But in contrast to that work, we used an explicit measure of prediction rather than tracking eye movements. We created four types of videos (see movies M1–M4) depicting cranberries moving from one container to another about 40 cm away. In a Hand-away video, a hand moved a cranberry from a container near the body to one farther away; in a Hand-toward video, a hand moved a cranberry from the far container to the near container. The No-hand videos were nearly identical except that the hand was digitally removed so that the cranberry appeared to move on its own. The participant's task was to press the down arrow key on the computer keyboard when the cranberry crossed the lip of the target container.

In the experiment, participants read blocks of 20 sentences. After each block of sentences, they viewed 20 videos, each depicting the transfer of one cranberry. The videos were comprised of a randomly ordered sequence of five Hand-away, five Hand-toward, five No-hand away, and five No-hand toward videos. Each of the five was a random selection from 10 videos of the same type. The reason for this random selection and random ordering was to prevent learning of the timing of particular cranberry movements.

Each participant read six blocks of 20 sentences (each followed by 20 videos). All of the sentences in a block were of the same type: sentences describing transfer away using the hand; sentences describing transfer toward using the hand; sentences describing transfer away using the leg (e.g., “You kicked the stone to Liam”); sentences describing transfer toward using the leg (e.g., “Liam kicked the stone to you”); and two blocks of 20 sentences that did not describe transfer events. The order of these six blocks was randomized for each participant.

During the sentence reading portions of the experiments, a participant read the sentence and judged whether it was written by a native speaker of English or a non-native speaker^2^. The point of this judgment was to focus the reader on each sentence. In addition, a randomly selected 25% of the sentences were followed by a four-alternative comprehension question. This question also was used to motivate processing of meaning and as a check that the participant was attending to the meaning.

If the MNS is adapted by the mere understanding of sentences presented before the videos, then prediction error (the time between when the cranberry actually crossed the lip of the container and the press on the computer key) should be greatest when the implied direction of the sentences (e.g., toward the reader) and the depicted direction of the cranberry movement are the same (Glenberg et al., 2008; Cattaneo et al., 2011). However, this effect should be greatest when the MNS is actively engaged, that is, when the video depicts biological motion as in the hand videos (cf. Flanagan and Johansson, 2003). Thus, for predictions following sentences describing transfer by hand, we predict a three-way statistical interaction between the implied direction of the sentence, the direction of cranberry movement, and whether the video shows a hand or not.

A different prediction is made for the predictions that follow blocks of sentences describing transfer by leg. Although the repeated simulation of these sentences should adapt leg action control, these adapted systems should not play a role in perceiving hand actions. Thus, the implied direction of movement in the leg sentences should not interact with the direction in the video, nor should there be an interaction with biological or non-biological movement.

## Methods^3^

### Participants

The study was approved by the Arizona State University IRB. The 90 participants (54 female) were university students, and all gave informed consent. All participants were native English speakers, right handed, and had normal or corrected-to-normal vision.

We used 20 triads of sentences with concrete objects that implied transfer by the hand. In addition, we constructed 20 triads of sentences in which the transfer was produced by the leg. An example of a leg triad is “Ethan bicycled the mail to you,” “You bicycled the mail to Ethan,” and “You read the mail with Ethan.” The sentences were arranged into six blocks (Hand Away, Hand Toward, Hand no-transfer, Leg Away, Leg Toward, and Leg no-transfer) of 20 sentences each. The order of the sentences within a block was randomized for each participant. The order of the blocks was randomized with the constraints that (a) no more than two hand or two leg blocks could occur successively and (b) two successive blocks could not both be Toward or Away.

For 5 of the 20 hand sentence triads and 5 of the 20 leg sentence triads we composed four-alternative multiple-choice questions about the content of the sentence. For example, for the sentence triad “Chloe danced the bouquet over to you,” “You danced the bouquet over to Chloe,” and “You smelled the bouquet with Chloe,” the multiple choice question was “What object was part of this event? (1) a car (2) a pencil (3) a flower (4) a window?” Thus, 25% of the sentences were followed by a comprehension question.

To create the videos, we began by filming 10 separate Hand-away videos and 10 separate Hand-toward videos. Each video began with a hand holding a cranberry above the start container for approximately 1 s. The hand then transferred the cranberry to the target container and dropped the cranberry. These videos were then digitally manipulated to produce 10 No-hand-away and 10 No-hand-toward videos. The manipulation used a masking procedure such that for each frame of the video everything was masked except for the location of the cranberry. These frames were then superimposed on a background similar to that in the original videos. The result was a video in which the cranberry appeared to move by itself and followed the exact path as in the corresponding Hand video. Following each block of sentences, participants observed a random selection of 20 videos with the constraint that there were exactly five of each type. The random selection and ordering of the videos made it difficult to use particular features (e.g., a slight pause in one video followed by a slight speeding in the next) to predict when the cranberry would cross the lip of the container. Consequently, we could collect more data from each participant without the worry that memory from previous trials was affecting the judgments.

### Procedure

Participants were informed that there would be six sections to the experiment, each consisting of two tasks: a sentence comprehension task and a visual prediction task. For the sentence comprehension task, the participant rested the right index finger on the “/” key and the left index finger on the “z” key. Participants were told that upon the presentation of a sentence, they were to judge whether the sentence was written by a native (“/”) or non-native (“z”) speaker of English (all were written by native English speakers). Furthermore, they were to use the 1–4 keys to answer the multiple-choice question if one occurred (after approximately every fourth sentence). For the video task, the participant was instructed to rest the right index finger on the “down arrow” key and to press the down arrow key when the cranberry crossed the lip of the target container.

Before the first block of sentences, participants practiced both tasks. For the sentence practice task, participants judged whether each of nine sentences was written by a native English speaker, and three of the nine were followed by multiple-choice comprehension questions. For the visual prediction practice task, participants watched a random selection of 12 videos.

## Results

The dependent variable was the difference (in ms) between the time when the cranberry first crossed the lip of the target container and when the participant pressed the down arrow key. However, we subjected the data to some pre-processing before conducting the analyses described below. First, we eliminated the data from 11 participants whose mean absolute prediction errors were more than two standard deviations from the mean^4^. Second, we intended to eliminate participants who answered the comprehension questions with less than 60% accuracy, however the two participants who met this criterion were already eliminated on the basis of their mean prediction errors. Finally, we noticed that two of the videos (a Toward Hand video and its paired No-hand video) had been inappropriately edited so that they were approximately twice as long as the other videos (the initial section of the video showing the hand above the start container was not edited down to 1 s). Data associated with these two videos were eliminated.

The prediction time errors were analyzed using multi-level modeling (the “mixed” procedure in SPSS). This procedure is similar to multiple regression in that regression coefficients corresponding to main effects of variables and their interactions are estimated. It is different from multiple regression in several regards, however. First, rather than using ordinary least squares to calculate the regression coefficients, they are estimated using maximum likelihood estimation (MLE). Second, the MLE procedure allows an estimate of the variance for each of the coefficients so that a *t*-test (*t* = coefficient/standard error) can be performed for each coefficient using its own error term. The calculation of the degrees of freedom in each variance makes use of the Satterthwaite estimation, and so the degrees of freedom often have a fractional component. Third, the multi-level modeling procedure allows the specification of multiple levels of dependency (and multiple random factors) that may correspond to the dependency of observations within subjects as different from the dependencies between subjects. Because separate variances are estimated for each coefficient, there is no need to ensure sphericity. Finally, the procedure has robust missing data handling so that we could use the data from a participant even if the participant did not respond to one or more cranberries. In the analyses, all predictor (independent) variables were centered.

Separate analyses were performed for predictions following Hand sentences and for predictions following Leg sentences. The predictors were the direction of the sentences read before the predictions (Toward or Away), the direction of transfer in the video (Toward or Away), and whether a hand was visible or not in the video.

**Figure 1:**
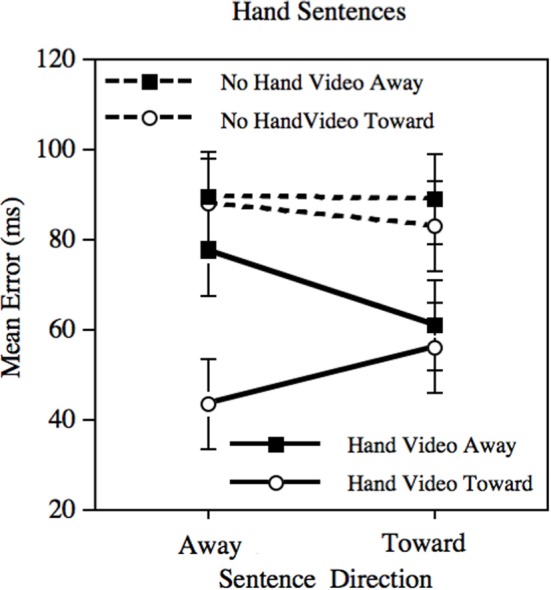
**Mean error in prediction following adaptation using the Hand sentences.** Error bars depict the standard error of the mean.

The mean prediction errors for the Hand sentences are presented in Figure 1. The predicted three-factor interaction was virtually significant (*p* = 0.054), *t*_(2895.26)_ = −1.93. Perhaps more importantly, when considering the Hand videos alone, the interaction between sentence direction and video direction was significant (*p* = 0.028), *t*_(1413.66)_ = 2.21. When considering the No-hand videos alone, the same interaction is not significant (*p* = 0.69). There were also several significant main effects, although none of theoretical interest: There were main effects of video direction (*p* < 0.004) and whether the video showed a hand or not (*p* < 0.001).

**Figure 2:**
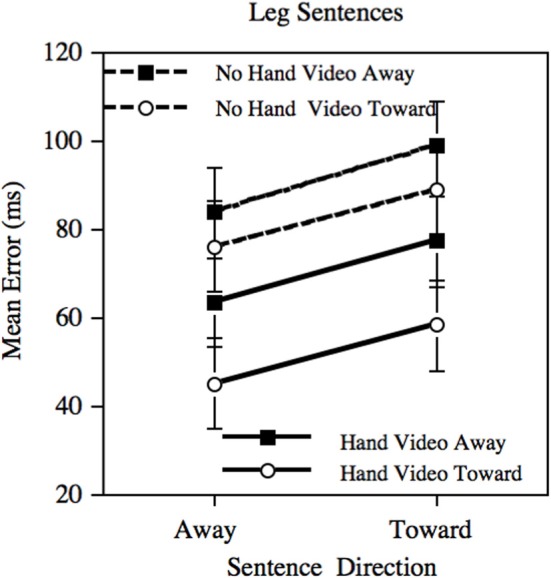
**Mean error in prediction following adaptation using the Leg sentences.** Error bars depict the standard error of the mean.

The mean prediction errors for the Leg sentences are presented in Figure 2. The three-factor interaction was not significant (*p* = 0.92); when considering the Hand videos alone, the interaction between sentence direction and video direction was not significant (*p* = 0.99); and when considering the No-hand videos alone, the same interaction was not significant (*p* = 0.88). There were, however, several significant main effects, although none of theoretical interest: There were main effects of sentence direction (*p* < 0.001), of video direction (*p* < 0.001), and whether the video showed a hand or not (*p* < 0.001).

## Discussion

Together with (Glenberg et al., 2008), these data demonstrate bi-directional adaptation effects between a component of the motor system, the MNS, and language That is, repeating literal action in one direction slows subsequent comprehension of sentences describing transfer in that same direction. And, as demonstrated here, comprehending sentences describing transfer in one direction disrupts subsequent perception of action in that same direction.

Several components of the results strongly suggest that they are caused by adapting the MNS. First, the effects are cross-modal. In Glenberg et al. (2008), adaptation using a motor task affected language comprehension. Here, adaptation using a language task (albeit conveyed through vision) affected visual predictions. Second, the effects are specific to an action goal (transfer Away or Toward) rather than a general priming or expectation effect. Third, the results reflect effector-specificity: only when the adapting linguistic stimulus implies transfer by hand is there an effect on predictions for the hand videos. Fourth, and most tellingly, the effects are only found when the prediction task involves a biological effector. That is, the MNS works through a process of motor resonance when the perceiver has goals similar to those accomplished by the perceived movements. If the perceived motion (e.g., No-hand cranberry motion) does not correspond to a motor action in the perceiver's motor repertoire, there should be little MNS involvement.

Caggiano et al. (2013) report that mirror neurons in macaque area F5 do not adapt to observation of repeated actions by changing their firing rate, thus suggesting that our results could not be due to adaptation of a MNS. However, Caggiano et al. also report that local field potentials in area F5, probably produced by input to the mirror neurons, do show adaptation. Thus, although we cannot claim that our procedure directly adapts mirror neuron activity, both our data and Caggiano are consistent with the claim that MNS activity as a whole is affected by adaptation.

These findings also suggest a constitutive relation between language comprehension and motor activity. Note that constitution is not a hypothesis that can be demonstrated by experiment. Experiments demonstrate causal relations, such as A causes B; constitution, however, is a particular form of causality, namely that A causes B because they are the same thing. How then do these results suggest constitution? The argument is one of parsimony. Namely, Glenberg et al. (2008) demonstrated a causal relation between adapting the motor system and language comprehension. Here we demonstrate the complement that using language as an adapting stimulus warps the MNS. Instead of having to propose two separate causal mechanisms, the notion that MNS activity constitutes (at least part) of language comprehension explains the results with a minimum of causal relations and mechanisms.

Nonetheless, it is important to keep in mind several limitations of our data and design. First, our data only support the notion of “bi-directional links” in a functional sense, and they do not demonstrate that the exact same pathways are active when action adapts language and when language adapts action systems. Second, transfer accomplished by the legs may not be as common as transfer accomplished with the hands. And finally, the case for bi-directionality would be stronger if we were to demonstrate that leg sentences would adapt prediction of leg videos.

Finally, we note that these data are not the first to demonstrate bi-directional causal effects between language and the motor system. Aravena et al. (2010) had participants read sentence implying hand actions with an open hand (e.g., applauding) or a closed hand (e.g., hammering). Upon understanding the sentence, the participant pressed a button using an open or closed hand. Then, using EEG, Aravena et al. found that an incompatible hand shape generated a larger N400-like component than a compatible hand shape. This finding implies a causal effect between motor preparation (hand shape) and semantics of the sentence. Aravena et al. also report that the implied hand shape in the sentence affected the motor potential (MP) component generated shortly before literal hand movement. This finding implies a causal effect between sentence comprehension and motor processes.

Guan et al. (2013) used a similar procedure to detect bi-directional links between the motor system and comprehension of abstract language. In particular, Guan et al. had participants read sentences that included the quantifiers “more and more” and “less and less.” On comprehending a sentence, the participant either moved the hand up to a response button (a direction compatible with “more and more”) or down to a response button (incompatible with “more and more”). Much like Aravena et al., Guan et al. also found a larger N400 for the incompatible trials and a larger MP in the compatible trials. Again, the results imply bi-directional links between language and motor processes.

Thus, subject to the limitations noted above, the data are strong in supporting the claim that there are bi-directional causal connections between aspects of language comprehension and the motor system. Furthermore, to the extent that the parsimony argument is correct, these bi-directional links suggest that motor activity constitutes at least a component of language comprehension (e.g., the understanding of human action). And finally, the data presented here support the claim that the MNS itself contributes to constitution.

### Conflict of interest statement

The authors declare that the research was conducted in the absence of any commercial or financial relationships that could be construed as a potential conflict of interest.

## Footnotes

^1^As will become apparent, we use the term “bi-directional” in the functional sense that the action system can affect language comprehension and language comprehension can affect the action system, and in both cases the affect is through the MNS. We do not mean that the exact same neurons or associations are themselves bi-directional.

^2^To create sentences that describe transfer, we used the double-object syntax, which strongly suggests transfer even for verbs not typically associated with transfer (Goldberg, 1995). For example, “You peddled the bike to Jace.” However, to get some of the leg sentences to strongly imply transfer, we needed to add the preposition “over to,” as in, “You jogged the bottle over to Olivia.” We used “over to” in half of the leg sentences. Consequently, we added “over to” to half of the Hand sentences, where the use of “over to” was not necessary but where it did not distract from the meaning, either, as in “Diane threw the pen over to you.” We used the Native English judgment to (a) focus the participant on each sentence and (b) justify what may appear to be an unnecessary use of “over to.”

^3^This reported experiment is the last in a series of three. Each experiment produced evidence consistent with a MNS explanation, but over the course of the experiments we learned better ways to test the claim. For example, in the initial experiment, we did not have a No-hand condition nor did we have leg sentences. Also, there are two important procedural details that differentiate the reported experiment form the first two experiments. First, in the initial experiments, participants judged if a sentence was sensible or not, and in fact, half of the sentences were intended to be nonsense. This procedure likely diluted any adaptation produced by simulating the sentences because half of the sentences were difficult or impossible to simulate. Second, in the initial experiments we presented the different types of cranberry actions in a continuous block, e.g., a block of 8 Hand-toward videos with no breaks in the filming or the responding. This procedure led to large effects of block order and large learning effects over the course of the experiment that then interacted with the effects of interest. By intermixing the different types of videos in the reported experiment, we avoided the learning effects and the block effects. Details regarding these initial experiments may be obtained from the corresponding author.

^4^As noted by a reviewer of a previous version of this article, some participants may have had difficulty seeing the rim of the container.
